# Comparison of culture‑negative and culture‑positive sepsis or septic shock: outcomes are more influenced by the nature of the infectious agent itself than by the samples’ positivity

**DOI:** 10.1186/s13054-021-03651-0

**Published:** 2021-08-12

**Authors:** Romain Jouffroy, Benoît Vivien

**Affiliations:** 1 Intensive Care Unit, Ambroise Paré Hospital, Assistance Publique – Hôpitaux de Paris and Paris Saclay University, Boulogne Billancourt, France; 2 Institut de Recherche bioMédicale et d’Epidémiologie du Sport - EA7329, INSEP - Paris University, Paris, France; 3 Centre de recherche en Epidémiologie et Santé des Populations - U1018 INSERM, Paris Saclay University, Boulogne Billancourt, France; 4 SAMU de Paris, Service d’Anesthésie Réanimation, Hôpital Universitaire Necker - Enfants Malades, Assistance Publique - Hôpitaux de Paris, and Paris University, Paris, France

**To the editor,**

Recently in the Journal, Li et al. [1] reported that culture positivity or negativity was not associated with differences in mortality, intensive care unit length of stay (LOS), mechanical ventilation requirements and renal replacement requirements of sepsis or septic shock patients. Conversely, hospital length of stay and mechanical ventilation duration of culture-positive septic patients were longer than those of culture-negative patients. While the authors must be congratulated for this systematic review and meta-analysis, we believe that their interpretation of the results requires caution. Firstly, we are surprised that for a major issue of interest such as blood culture positivity during sepsis, only seven studies were included by the authors in the final analysis. For example, the study by Vincent et al. [2], which is a 1-day, prospective, multicenter point reporting the prevalence of ICU infection, has not been included here, where 70% of their patients had positive microbial isolates. Secondly, despite international guidelines [3] on sepsis management, we cannot exclude heterogeneity between strategies and cares directly influencing outcomes. Thirdly, two studies [4, 5] include approximatively 19,000 patients, i.e., 85% of all cases, which could noticeably influence the results of this meta-analysis. Last but not least, we believe that more than the positivity or negativity of blood sampling, it is more the infectious agent itself that influences the outcomes, especially since the broad spectrum of the initial antibiotic therapy may be responsible for culture negativity.

Nevertheless, beyond all these limitations, we fully agree with Li et al. [1] that larger-scale studies are required to confirm or infirm their results.

## Data Availability

Not applicable.

## References

[1] Li Y, Guo J, Yang H, Li H, Shen Y, Zhang D (2021). Comparison of culture-negative and culture-positive sepsis or septic shock: a systematic review and meta-analysis. Crit Care.

[2] Vincent JL, Rello J, Marshall J, Silva E, Anzueto A, Martin CD, Moreno R, Lipman J, Gomersall C, Sakr Y, Reinhart K; EPIC II Group of Investigators. International study of the prevalence and outcomes of infection in intensive care units. JAMA. 2009;302(21):2323–9. 10.1001/jama.2009.1754 19952319

[3] Singer M, Deutschman CS, Seymour CW, Shankar-Hari M, Annane D, Bauer M, Bellomo R, Bernard GR, Chiche JD, Coopersmith CM, Hotchkiss RS, Levy MM, Marshall JC, Martin GS, Opal SM, Rubenfeld GD, van der Poll T, Vincent JL, Angus DC. The Third International Consensus Definitions for Sepsis and Septic Shock (Sepsis-3). JAMA. 2016. 23;315(8):801–10. 10.1001/jama.2016.0287 PMC4968574 26903338

[4] Kethireddy S, Bilgili B, Sees A, Kirchner HL, Ofoma UR, Light RB, Mirzanejad Y, Maki D, Kumar A, Layon AJ, Cooperative Antimicrobial Therapy of Septic Shock (CATSS) Database Research Group, et al. Culture-negative septic shock compared with culture-positive septic shock: a retrospective cohort study. Crit Care Med. 2018;46(4):506–12. 10.1097/CCM.0000000000002924 29293143

[5] Sigakis MJG, Jewell E, Maile MD, Cinti SK, Bateman BT, Engoren M (2019). Culture-negative and culture-positive sepsis: a comparison of characteristics and outcomes. Anesth Analg.

