# Supplementary material for: Comparison of culture‑negative and culture‑positive sepsis or septic shock: outcomes are more influenced by the nature of the infectious agent itself than by the samples’ positivity
Source: Crit Care. 2021 Aug 12;25:293. doi: 10.1186/s13054-021-03651-0 (PMC8359116; doi:10.1186/s13054-021-03651-0)
Supplement: Related texts

# Reply: Response to: Comparison of culture‑negative and culture‑positive sepsis or septic shock: outcomes are more influenced by the nature of the infectious agent itself than by the samples’ positivity

**Authors:** Yuting Li, Jianxing Guo, Hongmei Yang, Hongxiang Li, Yangyang Shen, Dong Zhang

**Affiliations:** grid.430605.4 Department of Intensive Care Unit, The First Hospital of Jilin University, Changchun, 130021 Jilin China

We would like to thank Dr. Jourffroy and Dr. Vivien for their interest in our manuscript [1] and for their comments in response to it. Mortality and other clinical outcomes between culture-negative and culture-positive septic patients have been documented inconsistently and are very controversial. That's why we performed a systematic review and meta-analysis to compare the clinical outcomes of culture-negative and culture-positive sepsis or septic shock.

Dr. Jourffroy et al. argued that the study by Vincent et al. [2] should be also included in the final analysis. However, as we all know that the most important principle of topic selection for meta-analysis is to have comparison. In this meta-analysis, we also clearly explained our inclusion and exclusion criteria as follows: (1) prospective and retrospective cohort studies; (2) patients with sepsis or septic shock; (3) all authors reported our primary outcome of all-cause mortality; (4) clearly comparing culture-negative versus culture-positive patients with clinically relevant secondary outcomes. We excluded studies including not estimable data and without clear comparisons of the outcomes. In addition, we excluded letter and review. The study by Vincent et al. is a 1-day, prospective, multicenter point reporting the prevalence of ICU infection. In other words, it is a cross-sectional study but not a cohort study, so it didn’t clearly compare culture-negative versus culture-positive patients with clinical outcomes. This study met our exclusion criteria but did not meet the inclusion criteria, that's why we didn't include it in the meta-analysis. We admit that the number of included cohort studies is small and we hope that there will be more studies in this area in the future.

Two included studies [4, 5] include approximatively 85% of all cases, which may noticeably influence the results of this meta-analysis. This has already been discussed in the limitation section of the article as follows: There was still substantial heterogeneity among the included cohort studies. Very heterogeneous populations were included in both retrospective and prospective cohort studies.

We agree with Dr. Jourffroy et al. that the infectious agent itself influences the outcomes of septic patients. We attempted to convey the message that culture positivity or negativity was not associated with mortality of sepsis or septic shock patients. A positive culture does not mean a severe infection, and a negative culture does not mean a mild infection. The clinical outcomes may be associated with not only the infection sources but also the management of the sepsis and septic shock.
